# Updates in the Diagnosis of Intraductal Neoplasms of the Pancreas

**DOI:** 10.3389/fphys.2022.856803

**Published:** 2022-03-04

**Authors:** Naziheh Assarzadegan, Sepideh Babaniamansour, Jiaqi Shi

**Affiliations:** Department of Pathology, University of Michigan, Ann Arbor, MI, United States

**Keywords:** intraductal papillary mucinous neoplasm, intraductal oncocytic papillary neoplasm, intraductal tubulopapillary neoplasm, pancreatic ductal adenocarcinoma, classification

## Abstract

Pancreatic ductal adenocarcinoma (PDAC) is one of the deadliest types of cancer worldwide. There are many reasons for this dismal prognosis, including the advanced stage at the time of diagnosis and the lack of effective therapeutic approaches. Intraductal papillary mucinous neoplasms (IPMNs) represent detectable and treatable precursor lesions of PDAC. Our understanding of the pathology of IPMNs has evolved over the past few decades, and new advances in diagnostic tools have emerged. The new World Health Organization (WHO) classification scheme now recognizes the previously considered variants of IPMNs, such as intraductal oncocytic papillary neoplasms (IOPNs) and intraductal tubulopapillary neoplasms (ITPNs), as distinct neoplasms. New imaging and molecular diagnostic tests are being developed to recognize these PDAC precursor lesions better. Here, we review the advances in diagnostic tools for IPMNs, IOPNs, and ITPNs, emphasizing the new (5th edition, 2019) WHO classification for pathological diagnosis, molecular markers, new laboratory tests, and imaging tools.

## Introduction

All cystic tumors of the pancreas were grouped decades ago. In 1978, Compagno et al. separated mucin-producing neoplasms of the pancreas from serous cystadenomas (Compagno and Oertel, 1978a,b; Hodgkinson et al., 1978). In 1980, Ohhashi et al. (1982) first described what we now recognize as intraductal papillary mucinous neoplasms (IPMNs). A few years later, Zamboni and others separated IPMNs from mucinous cystic neoplasms (MCNs; Zamboni et al., 1999).

Intraductal papillary mucinous neoplasms are precursor lesions of pancreatic ductal adenocarcinoma (PDAC), which has a dismal prognosis. PDACs predominantly arise from two types of precursor lesions, pancreatic intraepithelial neoplasia (PanIN), and IPMNs. PanINs are considered microscopic lesions (usually <5 mm) and are not typically detected radiologically. In contrast, IPMNs lead to cystic dilatation of the pancreatic duct, which imaging studies can readily identify. IPMNs are the intraductal proliferation of mucinous epithelial cells, characterized by abundant mucinous production and papillary epithelial growth. As a result of detailed studies of the pathologic and genetic features of IPMNs in recent years, previously considered variants of IPMN, intraductal oncocytic papillary neoplasms (IOPNs), and intraductal tubulopapillary neoplasms (ITPNs) are now regarded as distinctive neoplasms as per the 2019 5th edition of World Health Organization (WHO) classification scheme (WHO Classification of Tumours Editorial Board, 2019).

Intraductal papillary mucinous neoplasms of the pancreas originate from the pancreatic ductal system. Depending on the pancreatic ductal sites affected, IPMNs can be divided into three groups: (a) main-duct IPMNs (MD-IPMN), which originate from the main pancreatic duct; (b) branch-duct IPMNs (BD-IPMN), which originate from the ductal branches of the main duct; and (c) mixed IPMNs which originate from both the main and side-branches of the ductal system (Salvia et al., 2004; Rodriguez et al., 2007). Depending on the level of epithelial dysplasia, IPMNs are further classified into low grade and high grade. IPMNs have been shown to progress from low grade to high grade and eventually to invasive carcinoma, which can be colloid or an invasive ductal adenocarcinoma (Shimizu et al., 2013; WHO Classification of Tumours Editorial Board, 2019). The overall incidence of an invasive carcinoma associated with IPMN is low; however, they are seen in association with about 60% of MD-IPMNs.

Pancreatic ductal adenocarcinoma can occur in the vicinity of IPMN lesions. The term concomitant PDAC with IPMN refers to a PDAC that is separated from the IPMN by an uninvolved segment of pancreatic duct and with no areas of transition in between. The frequency of concomitant pancreatic cancer in IPMN patients range from 2.5 to 9.2% by various studies. PDAC concomitant with IPMN are often significantly smaller, less aggressive, and are associated with longer survival compared to PDAC with no associated IPMN (Yamaguchi et al., 2011; Tanaka, 2015).

Intraductal oncocytic papillary neoplasms, previously known as an oncocytic variant of IPMNs, are rare cystic neoplasms with intraductal growth. It was recently discovered that virtually all IOPNs harbor specific fusion genes not found in any other intraductal neoplasms, which helped establish that IOPNs should be considered an entirely separate entity from the other IPMNs. Invasive adenocarcinoma is seen in about 30% of IOPNs (Basturk et al., 2016; Vyas et al., 2020).

Intraductal tubulopapillary neoplasms is another rare intraductal neoplasm distinct from IPMN due to the lack of mucin production, uniform high-grade nuclear atypia, and tubule formation. They are often less cyst forming than IPMNs. Invasive carcinoma is found in association with 70% of these lesions (Basturk et al., 2017).

In the last decade, advances in understanding IPMNs, IOPNs, and ITPNs have enabled early diagnosis and provided preoperative risk measurements to prioritize those patients who will benefit the most from surgical resection. This article will provide a brief overview of the advances of the diagnostic tools for IPMNs, IOPNs, and ITPNs, emphasizing the 5th edition of WHO classification for pathological diagnosis, molecular markers, new laboratory tests, and imaging tools.

## Imaging Tools

Cross-sectional and ultrasonographic imaging modalities used to diagnose and assess IPMNs have their own merits and limitations. Generally, imaging studies have two primary goals in the evaluation of IPMNs. Firstly, imaging studies aim to distinguish IPMN from other types of pancreatic cysts. Secondly, radiologic investigations aim to recognize the involvement of the main pancreatic duct to assess the risk of IPMN progressing to high-grade dysplasia or invasive carcinoma. Another marker used for the risk assessment of IPMN is the presence of mural nodules within the pancreatic cyst or adjacent to it. Preoperative radiological investigations are critical for identifying the malignancy risk in IPMNs. In 2012, the Fukuoka guidelines recommended considering the “high-risk stigmata” and “worrisome features” as criteria for the immediate surgical resection of IPMN (Tanako et al., 2012). Radiological and clinical findings in patients with IPMNs with high-risk stigmata include dilated main pancreatic duct (≥10 mm), obstructive jaundice, and enhanced solid component. The worrisome features on endoscopic ultrasound (EUS) examinations include large cyst (≥3 cm), thickened and enhanced cyst walls, abrupt dilatation of main pancreatic duct (5–9 mm), distal pancreatic atrophy, non-enhancing mural nodules, and lymphadenopathy (Hecht et al., 2021).

Magnetic resonance with cholangiopancreatography (MRCP) and multidetector computed tomography (MDCT) are reliable and effective imaging modalities for assessing pancreatic cyst size, morphology, and communication within the main pancreatic duct. Both MRCP and MDCT are estimated to distinguish between mucinous and non-mucinous cysts with 71–85% accuracy. MDCT, particularly dual-phase pancreatic protocol CT and gadolinium-enhanced MRI, is effective for the initial assessment of suspected IPMN. Both modalities have a 75–86% accuracy in identifying lesions with high-grade dysplasia and invasive carcinoma (Kitano et al., 2011; Morales-Oyarvide et al., 2017).

Magnetic resonance with cholangiopancreatography is believed to be more accurate than computed tomography (CT) for evaluating pancreatic cysts. It allows superior visualization of the anatomy of the ductal system and enables the detection of internal septations and mural nodules. MRCP has a 91–100% sensitivity and a specificity of 89% for assigning communication with the main pancreatic duct (Sainani et al., 2009). A CT scan is indicated in patients suspected of metastatic disease and patients who may have a postoperative recurrence of PDAC. A CT scan can assist in detecting calcifications and help assess vascular involvement.

Endoscopic ultrasound is highly accurate in evaluating the pancreatic parenchyma and the cystic component. It is an effective modality for detecting mural nodules. For the evaluation of mural nodules, contrast harmonic enhanced EUS is superior to standard EUS as it has a specificity of 80% and a sensitivity of 100% (Alvarez-Sánchez and Napoléon, 2014). Contrast-enhanced harmonic EUS allows detection of blood flow in the suspected mural nodule and can help distinguish true mural nodules from mucin plugs with a sensitivity of 89–96% and a specificity of 64–88% (Kitano et al., 2011). Furthermore, EUS-guided fine-needle aspiration (FNA) allows cytological sampling of any solid component (Kitano et al., 2011). It can also be used for aspirating cyst fluid for subsequent protein, metabolite, molecular, and cytologic evaluation.

Different endoscopic modalities can be used to characterize suspected IPMN. Patulous “fish mouth” papilla extruding mucus is a pathognomonic feature of IPMN involving the main pancreatic duct. This classic sign can be observed *via* upper endoscopy (Salvia et al., 2004). Endoscopic retrograde cholangiopancreatography with or without pancreatoscopy can help obtain pancreatic juice and brushings for cytology. It can also assist in the visualization of villous or papillary growths along the ducts.

## Updates in the 5th Edition of WHO Classification for Pathological Diagnosis of Pancreatic Intraductal Neoplasms

As mentioned earlier, because of the integrated understanding of pathological and genetic features of the pancreatic intraductal neoplasms, in the revised WHO classification scheme, IOPN and ITPN are now considered distinct neoplasms and not variants of IPMN. Also, the most recent WHO guidelines have simplified the dysplasia grading system. The dysplasia grading system has been simplified from a three- to now two-tiered grading system for these neoplasms: low-grade and high-grade dysplasia. The previously designated “intermediate-grade dysplasia” is now included in low-grade dysplasia. Low-grade neoplasms have mild to moderate atypia, well-oriented nuclei without pseudostratification, simple papillae, and rare mitoses. In contrast, high-grade neoplasms have marked atypia, complex architecture with nuclear stratification, loss of nuclear polarity, and mitoses that are brisk (WHO Classification of Tumours Editorial Board, 2019). If a neoplasm contains both low-grade and high-grade dysplasia, the highest grade is assigned to the neoplasm. The presence of high-grade dysplasia has significant clinical implications. High-grade neoplasms are at a higher risk of having an associated invasive carcinoma, and the presence of high-grade dysplasia in a surgically resected specimen is a significant risk factor for recurrence in the remnant pancreas. These updates are summarized in Table 1.

**Table 1:** Summary of 5th edition of WHO updates compared to the previous edition (4th edition).

WHO 4th Edition	WHO 4th Edition
Oncocytic variant of IPMN	IOPN is now considered a separate entity
ITPN was considered a variant of IPMN	ITPN is now considered a separate entity
Three-tiered dysplasia grading system Low-grade Intermediate High-grade	Two-tiered dysplasia grading system Low-grade (original intermediate grade is part of the spectrum of low-grade dysplasia) High-grade

## Gross Features

Intraductal papillary mucinous neoplasms are grossly visible (>5 mm) intraductal epithelial neoplasms with excessive mucin production. MD-IPMNs are typically found in the pancreatic head but can also involve the entire main pancreatic duct. BD-IPMNs mainly occur in the uncinate process as peripheral multicystic lesions in an otherwise unremarkable pancreatic parenchyma. It has been shown by various studies that MD-IPMNs are significantly more likely to harbor high-grade dysplasia or have an associated invasive carcinoma than the BD-IPMNs (Rodriguez et al., 2007; Hirono et al., 2017; Attiyeh et al., 2018). This finding has important clinical implications as MD-IPMNs and BD-IPMNs can often be distinguished by imaging modalities, which provide a preoperative risk assessment for patients who will benefit from surgical resection. Therefore, because of the higher likelihood of high-grade dysplasia or an associated invasive carcinoma, most MD-IPMNs are treated with surgical resection, while most asymptomatic BD-IPMNs can be followed without surgical intervention. Mural nodules can also be found in some IPMNs. Compared to the flatter areas of an IPMN, mural nodules are more likely to have epithelium with high-grade dysplasia (Adsay et al., 2016). In addition, IPMNs more than 30 mm are more likely to have high-grade dysplasia and associated invasive carcinoma (WHO Classification of Tumours Editorial Board, 2019). These features are clinically significant as they can also provide preoperative risk assessments for those patients who will likely benefit from surgical resection. It is estimated that 20–40% of IPMNs are grossly multifocal. (Matthaei et al., 2012) The multifocality of IPMNs can help differentiate them from mucinous cystic neoplasms, which are often unifocal.

Intraductal oncocytic papillary neoplasms are often large (average size of 5.5 cm) intraductal solid nodules or papillary projections with pancreatic duct dilatation but less intraductal mucin accumulation (WHO Classification of Tumours Editorial Board, 2019).

Intraductal tubulopapillary neoplasms are often solid, not cystic, rubbery, or fleshy, nodular lesions in the dilated pancreatic ducts with an average size of 4.5 cm. They typically lack mucin secretion (WHO Classification of Tumours Editorial Board, 2019).

## Histopathology

**Figure 1:**
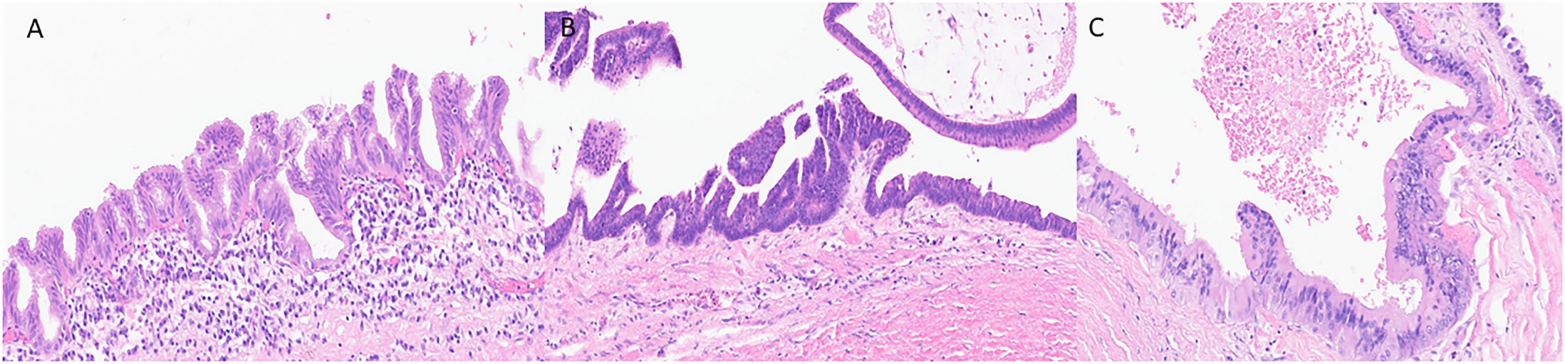
Representative histological pictures of intraductal papillary mucinous neoplasm (IPMN). **(A)** IPMN with gastric-foveolar differentiation. The epithelium is flattened with basally placed nuclei and abundant mucin cap resembling gastric-foveolar epithelium. **(B)** An IPMN with intestinal differentiation forming villous papillae resembling villous adenoma of the colon. The cells have basophilic cytoplasm with enlarged oval and hyperchromatic nuclei and scattered goblet cells. **(C)** IPMN with pancreatobiliary differentiation. The neoplastic cells are cuboidal, have enlarged nuclei with amphophilic cytoplasm. **(A–C)** Original magnification 200×.

Histologically, the neoplastic epithelium of IPMNs can have various directions of differentiation. The most seen differentiations include gastric-foveolar, intestinal, and pancreatobiliary (Figure 1). However, IPMNs can display a combination of different differentiation. Gastric-foveolar differentiation is the most common type and usually occurs in BD-IPMNs. The neoplastic epithelium usually forms broader and thicker papillae and is composed of columnar epithelium with basally placed nuclei and overlying mucin caps resembling gastric-foveolar epithelium (Figure 1A). The epithelium could be flat and typically has only low-grade dysplasia (Ban et al., 2006).

Intraductal papillary mucinous neoplasm with intestinal differentiation is the second most common type in which the neoplastic epithelium forms long finger-like (villous) papillae (Figure 1B). The neoplastic cells show tall-columnar cells with elongated hyperchromatic nuclei. Pseudostratification with high-grade dysplasia is often seen. All colloid carcinomas of the pancreas arise from an intestinal-type IPMN (Adsay et al., 2004; Furukawa et al., 2005).

The neoplastic epithelium forms complex, thin, and branching papillae in IPMNs with pancreatobiliary differentiation, the least common form. The cytoplasm in these cells is amphophilic, and they have enlarged nuclei and often prominent nucleoli (Figure 1C). The neoplastic cells in IPMNs with pancreatobiliary differentiation are more cuboidal (less columnar) than those seen in other IPMN differentiation types. These often have high-grade dysplasia (Adsay et al., 2002; Furukawa et al., 2005).

As delineated earlier, a two-tiered grading system (low- vs. high grade) is now used to assign the degree of dysplasia to these neoplasms. Studies have shown that the grade of dysplasia has more clinical importance than the direction of epithelial differentiation. High-grade neoplasms are more likely to have an associated invasive carcinoma. The presence of high-grade dysplasia in surgically resected neoplasm is associated with a higher risk of progression in the remnant pancreas following surgery (Rezaee et al., 2016; Amini et al., 2020).

**Figure 2:**
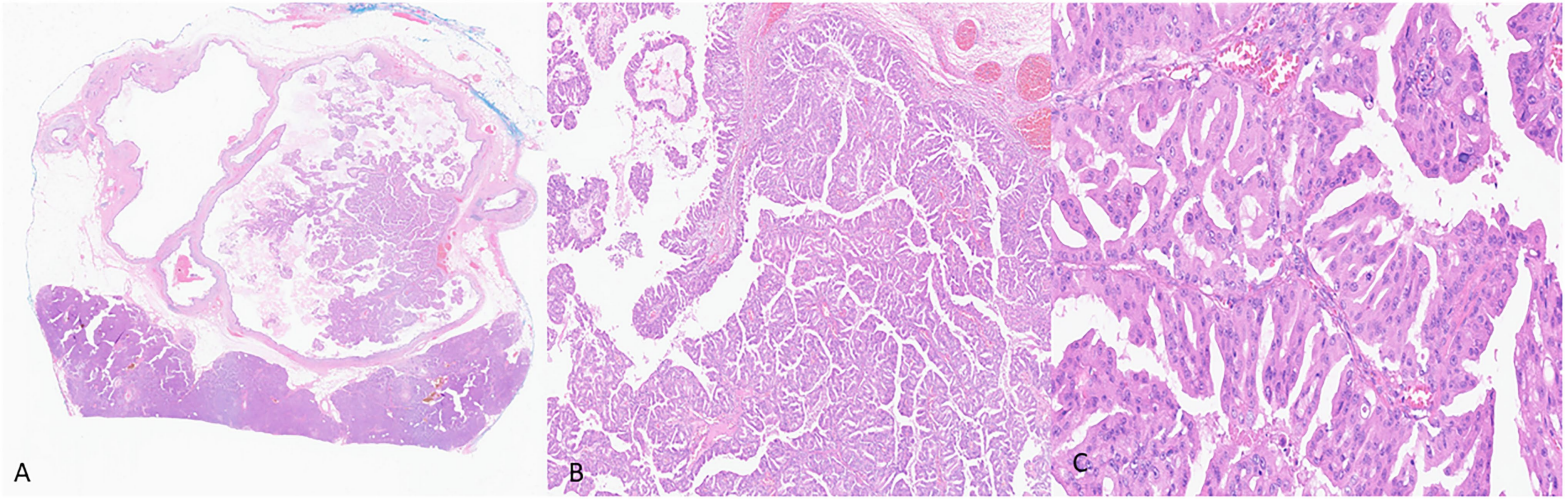
Representative histological pictures of intraductal oncocytic papillary neoplasm (IOPN). **(A)** Low magnification shows intraductal papillary proliferation. **(B)** Intermediate magnification shows the thick, complex papillae. **(C)** Higher magnification shows that the neoplastic cells have abundant and distinctly oncocytic (eosinophilic) cytoplasm. The nuclei are round and often contain prominent nucleoli. **(A)** Original magnification 10×. **(B)** Original magnification 100×. **(C)** Original magnification 200×.

In IOPNs, the neoplastic epithelium forms complex, thick papillae with intraepithelial and intracellular lumina (Figure 2). The neoplastic cells contain abundant eosinophilic cytoplasm, and the nuclei are round with prominent nucleoli. IOPNs almost always have high-grade dysplasia and are associated with invasive carcinoma in about 30% of the cases (WHO Classification of Tumours Editorial Board, 2019). Rarely, the invasive component may show abundant mucin accumulation (Wang et al., 2019). IOPNs with a predominantly solid pattern can resemble acinar cell carcinoma or pancreatic neuroendocrine neoplasms. Immunohistochemistry for BCL-10, trypsin, and neuroendocrine markers is essential in this differential diagnosis.

**Figure 3:**
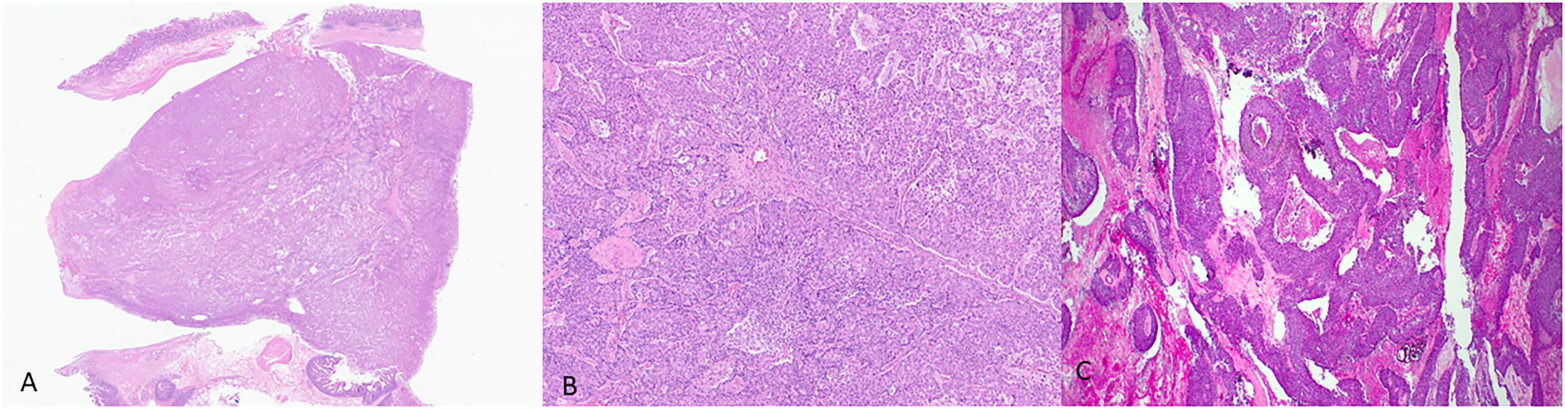
Representative histological pictures of intraductal tubulopapillary neoplasm (ITPN). **(A)** Low magnification shows small, tightly packed glands forming intraductal nodules. **(B)** Higher magnification shows the cuboidal neoplastic cells with no significant mucin. **(C)** Another case of ITPN showing foci of comedo-type necrosis resembling intraductal carcinoma of the breast. **(A)** Original magnification 10×. **(B)** Original magnification 200×. **(C)** Original magnification 100×.

Histologically, ITPNs are composed of tightly packed glands and tubules, forming large intraductal nodules with smooth contours (Figure 3). The neoplastic cells are cuboidal, with a moderate amount of amphophilic to eosinophilic cytoplasm, and they typically do not contain intracellular mucin. Invasive carcinoma is seen in 70% of the cases but is usually limited in extent. It is often difficult to determine the presence of an invasive carcinoma as many of the neoplastic nodules lack a peripheral rim of non-neoplastic epithelium. It is often helpful to look for invasion in areas away from the periphery of the nodules and search for individual cells and angulated small glands that are embedded in a desmoplastic stroma (Yamaguchi et al., 2009; Basturk et al., 2017). The differential diagnosis of ITPN includes acinar cell carcinoma, which may have an intraductal growth pattern. Unlike ITPNs, acinar cell carcinomas have apical acidophilic granules and intraluminal secretions. Immunohistochemistry for BCL-10 and trypsin can help in this differential.

**Figure 4:**
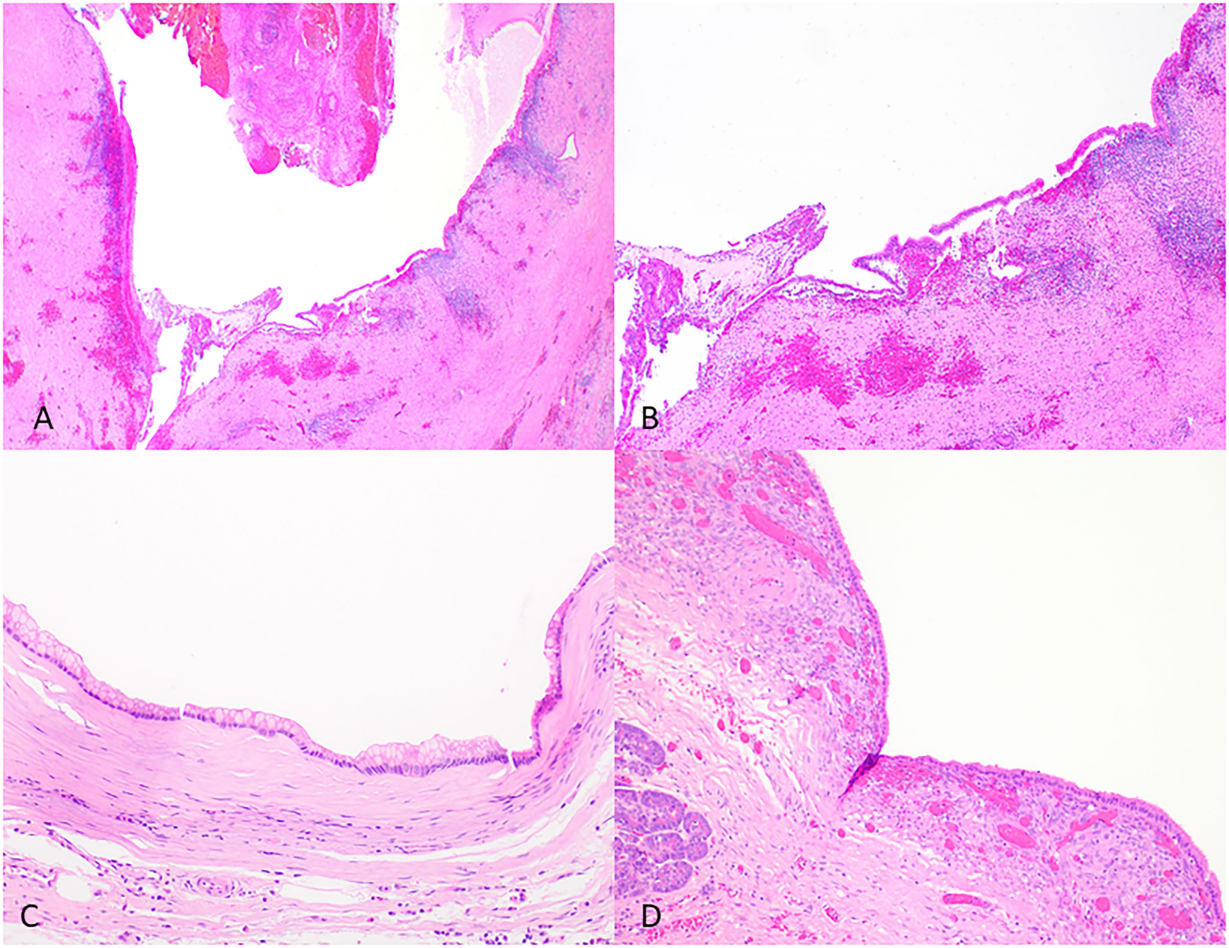
Representative histological pictures of retention cyst, simple mucinous cyst, and mucinous cystic neoplasm. **(A)** Retention cyst. Low magnification shows a dilated pancreatic duct. **(B)** Higher magnification shows a flattened epithelium with no papillary projections. Note the background pancreas with extensive atrophy and fibrosis. No ovarian-type stroma is seen. **(C)** Simple mucinous cyst. The cyst is lined by a benign mucinous epithelium and lacks an ovarian stroma. **(D)** Mucinous cystic neoplasm. The cyst is lined by mucinous epithelium and unlike simple mucinous cyst or a retention cyst is associated with an ovarian-type stroma. **(A)** Original magnification 10×; **(B)** Original magnification 100×; and **(C,D)** Original magnification 200×.

The differential diagnosis of IPMNs includes mucinous cystic neoplasms (MCNs), other intraductal neoplasms, simple mucinous cysts, and retention cysts (Figure 4 and Table 2).

**Table 2:** Differential diagnosis of IPMN.

	Intraductal papillary mucinous neoplasm	Mucinous cystic neoplasm	Retention cyst	Simple mucinous cyst
Demographics	More common in elderly men (7th–8th decade of life)	More common in women (5th decade)	F = M Any age	F = M Any age
Location	Often head of the pancreas (>80%)	Tail or body (>90%)	Anywhere in the pancreas	Anywhere in the pancreas
Imaging	Dilated main pancreatic duct or multilocular cyst Mural nodules may correspond to invasive carcinoma or high-grade dysplasia	Multilocular or unilocular thick-walled cyst No connection with the main pancreatic duct	Unilocular cyst	Unilocular cyst
Histology	Papillary projections With low-grade or high-grade dysplasia	No connection with the main duct Contain ovarian-type stroma (ER+, PR+)	Downstream obstruction Lined by flattened epithelium, and lack papillary projections	No obstructive process No papillary projections No ovarian-type stroma
Molecular	*KRAS* *GNAS* *TP53/PIK3CA/PTEN* *RNF43* *BRAF* *KLF4* *P16/CDKN2A* *SMAD4* *TGFBR2*	*KRAS* *SMAD4* *TP53*	No specific mutations	*KRAS* *KMT2C* *BRAF* *RNF43* *CDKN2A* *SMAD4* *TP53*
Cyst fluid analysis	Elevated CEA levels (of >200 ng/ml) High amylase level	Elevated CEA Low amylase	Variable CEA or amylase	Variable CEA or amylase

Retention cysts are caused by dilatation of the pancreatic ducts due to a downstream obstructive process. They are often unilocular and lined by a flattened epithelium and lack the florid papillary projections of IPMNs (Figure 4). If no obstructive process is present, mucinous cysts larger than 1 cm and do not have characteristic histologic features of IPMN can be classified as simple mucinous cysts (Krasinskas et al., 2017). MCNs typically occur in women, almost always located in the tail or body, and do not communicate with the duct system. They contain ovarian-type stroma that can be demonstrated by immunohistochemistry staining of PR and ER (Wilentz et al., 2000; Figure 4).

## Immunohistochemical Markers

While immunolabeling can be used in identifying the various types of differentiation in IPMNs, this distinction is not as clinically significant as the degree of dysplasia. Most IPMNs are labeled with ductal markers, including pancytokeratin (AE1/AE3), cytokeratins-7, 8, 19, and CEA. All types of IPMN express MUC5AC. IPMNs with intestinal differentiation are labeled with antibodies to cytokeratin 20 and CDX2. IPMNs with pancreaticobiliary differentiation mark with MUC1 and IPMNs with gastric-foveolar differentiation show variable expression with MUC6. For IOPNs, the immunohistochemical markers expressed include EMA (MUC1), MUC6, hepatocyte-1 (Hep Par-1), and CD117 (Mattiolo et al., 2020). MUC2 and MUC5AC expression are limited to goblet cells. ITPNs are most commonly labeled with MUC1 and MUC6, while consistently negative for MUC5AC (WHO Classification of Tumours Editorial Board, 2019). The various immunophenotypes of these neoplasms are summarized in Table 3.

**Table 3:** Immunohistochemical profile of IPMNs, IOPNs, and ITPNs.

IPMN	Cytokeratins	CK20	MUC1	MUC2	MUC5AC	MUC6	CDX2
Gastric	+	−	−	−	+	+/−	−
Intestinal	+	+	−	+	+	−	+
Pancreatobiliary	+	−	+	−	+	+	−
IOPN	+	+ in GCs	+	+ in GCs	+	+	+ in GCs
ITPN	+	−	+	−	−	+	−

GC, goblet cells.

## Genetic Alterations

The new advances in molecular studies have revolutionized our understanding of human neoplasms, including IPMNs. Various genes have been identified as the driver genes following exome sequencing of IPMNs. Somatic activating mutations in the *KRAS* and *GNAS* genes are the most common alterations (Wu et al., 2011). Other less common alterations include *KLF4, PIK3CA, p16/CDKN2A, RNF43, SMAD4, TGFBR2, TP53,* and rarely *BRAF* mutations (Schonleben et al., 2006; Fujikura et al., 2021) Gene fusions such as *ATP1B1-PRKA*CB and *DNAJB1-PRKACA* are restricted to IOPNs (Singhi et al., 2020; Vyas et al., 2020). Germline genetic alterations have also been reported in patients with IPMNs. In a study by N. Roberts and colleagues, 23 (7.3%) of the 315 patients who had surgically resected IPMNs showed deleterious variants in *ATM, PTCH1,* and *SFU* genes (Skaro et al., 2019). Biallelic inactivation of the *STK11* gene has been reported in IPMNs in patients with Peutz-Jeghers syndrome (Sato et al., 2001). Identifying these genetic alterations can be a valuable tool in studying these lesions, and potentially, they can have diagnostic implications and increase diagnostic accuracy. For example, while studying IPMNs, Wood et al. showed that the different foci of IPMN in the same pancreas harbor different somatic alterations, which confirms the multifocality of IPMNs (Pea et al., 2017; Fischer et al., 2019).

Distinct patterns of methylation have been reported in IPMNs. Methylations of *BNIP3, CDO1, EBF3, NXPH1, PTCHD2, and SOX17* are most reported in high-grade IPMNs than low-grade IPMNs (Hong et al., 2012; Fujiyama et al., 2020). Methylation profiling is now practiced for brain tumor classification and is emerging for pancreatic neuroendocrine neoplasms and has the potential to be used in EUS-FNA cyst fluid as an adjunct tool in further classifying the cyst type.

MicroRNAs are currently recognized as biomarkers and molecular targets of various diseases, including malignancies. RNA markers can also be used as a diagnostic tool in the EUS-FNA obtained cyst fluid. Approximately 80% of IPMNs show high expression for microRNAs—miR-155 and miR-21. The expression of these microRNAs is particularly noticeable in advanced IPMNs and has been investigated as a potential diagnostic approach in pancreatic cyst fluid as a marker for evaluating the malignant transformation of IPMNs (Shirakami et al., 2021).

The use of microRNAs in different types of body fluids such as urine, serum, or saliva has also been studied as biomarkers for the early detection of pancreatic cancer (Ishige et al., 2020). For example, urine miR-30e, miR-143, miR-223, and miR-204 levels and serum miR-21 and miR-34a have been proven useful as potential minimally invasive biomarkers for the diagnosis of PDAC (Debernardi et al., 2015; Alemar et al., 2016). The combination of urine miR-143 with miR-30e achieved a sensitivity of 83.3% and a specificity of 96.2% for diagnosing pancreatic cancer in one study (Debernardi et al., 2015). The sensitivity and specificity of miR-21 were 82.6 and 77.8%, and those of miR-34a were 91.3 and 77.8% for discriminating PDAC from control samples, respectively (Alemar et al., 2016). Combining both serum and urine miR-1246 levels yielded a sensitivity of 85% for the diagnosis of pancreatic cancer (Ishige et al., 2020). The expression of hsa-miR-21, hsa-miR-23a, hsa-miR-23b, and miR-29c is significantly upregulated in the saliva of pancreatic cancer patients compared to control, showing sensitivities of 71.4, 85.7, 85.7, and 57%, respectively, and 100% specificity (Humeau et al., 2015). Salivary miR-3679-5p and miR-940 also show good discriminatory power to detect respectable pancreatic cancer when compared to healthy controls, with reasonable specificity (45 and 40%) and sensitivity (82.5 and 90%; Xie et al., 2015).

## Cyst Fluid Analysis and Cytopathology

Cyst fluid can be characterized by protein analysis, metabolite analysis, cytological analysis, and molecular analysis. Cyst fluid protein analysis includes CEA, mAB-Das 1, and amylase (Cizginer et al., 2011; Jhala et al., 2020; Das et al., 2021). Carcinoembryonic antigen (CEA) is the most studied protein marker in pancreatic cyst fluid (Cizginer et al., 2011). Although CEA cannot effectively distinguish between benign cysts and those with high-grade dysplasia or invasive carcinoma, it can effectively differentiate between mucinous and non-mucinous lesions. A cyst fluid CEA threshold value of 192 ng/ml is the most widely used cut-off value. A value of >200 ng/ml strongly supports a neoplastic mucin-producing cyst. However, it does not help differentiate IPMN from MCN or low-grade from high-grade IPMN. mAB-Das 1 is a monoclonal antibody that reacts specifically with intestinal metaplasia of the lower esophagus and normal colonic epithelium. This antibody has been found to detect high-risk IPMN with a sensitivity of 89% and specificity of 100% (Das et al., 2021). High amylase levels in cyst fluid can confirm communication with the main pancreatic duct and suggest either a pseudocyst or IPMN. CA 19.9 can be a useful biomarker for distinguishing mucinous and non-mucinous lesions when CEA values are indeterminate. The cytopathologic evaluation confirms the diagnosis and can provide the degree of dysplasia. IPMNs often show thick inspissated mucin and papillary projections, which are highly characteristic when evident. The sensitivity of the cytological analysis can be hampered because of the scant cellularity, the heterogeneity of the lesion, insufficient aspirated volume, and contamination of the fluid with gastric and duodenal mucosal cells.

Molecular analysis on EUS-FNA obtained cyst fluid is increasingly employed and can enhance the diagnostic accuracy when used in combination with protein markers such as VEGFA and CEA (Cizginer et al., 2011; Carr et al., 2017). Mutations in *KRAS* and *GNAS* are the two common gene alterations seen in IPMN (Schmitz et al., 2021). *KRAS* mutations are highly specific to mucinous lesions and are found in the cyst fluid of more than 50% of IPMN cases (Nikiforova et al., 2013; Singhi et al., 2018). Singhi et al. (2018) found that the presence of *TP53/PIK3CA/PTEN* mutations was indicative of high-grade dysplasia. Therefore, sequencing the cyst fluids can be a highly sensitive and specific tool for diagnosing the cyst type. *GNAS* gene alterations are believed to be specific to IPMNs and are observed in the fluid of approximately two-thirds of cases (Wu et al., 2011; Kadayifci et al., 2017). However, gene mutations do not indicate the risk of high-grade dysplasia or invasive carcinoma. While these molecular advances are promising when combined with clinical features, they suffer from imperfection due to the underlying heterogeneity of IPMNs.

Recently, we have reported that specific metabolites in the cyst fluid can also be used as potential diagnostic biomarkers to distinguish malignant from benign pancreatic cysts and mucinous from non-mucinous cysts (Shi et al., 2021). We found that (Iso)-butyrylcarnitine alone has a diagnostic accuracy of 89% to separate malignant from benign pancreatic cysts, and 5-oxoproline alone has a diagnostic accuracy of 90% to differentiate mucinous from non-mucinous cysts (compared to previously reported glucose which has an accuracy of 82% in our study; Park et al., 2013).

## Clinical Relevance

Advances in our understanding of the pathology and molecular features of IPMNs, IOPNs, and ITPNs have significant clinical implications. Importantly, IPMNs are heterogeneous neoplasms and can be multifocal. It is not practically possible to sample all the foci. Hence, the preoperative cyst fluid analysis and cytologic evaluation, while accurate for the sampled focus, are inherently imperfect, particularly regarding the degree of dysplasia. As a manifestation of IPMN multifocality, there is a risk of synchronous or metachronous disease in the remaining portion of a patient’s pancreas in those who have undergone surgery. The risk is higher in MD-IPMNs and those with high-grade dysplasia. Therefore, careful clinical follow-up is necessary to rule out synchronous or metachronous disease in the remnant pancreas (He et al., 2013). If not initially performed, it would also be important to submit the entire lesion for histologic evaluation to exclude the presence of invasive carcinoma.

Another issue of clinical concern is whether IPMN at the resection margin is associated with the risk of progression or recurrence. However, the studies are controversial. While some data have shown that the presence of high-grade dysplastic focus of >0.5 cm at the resection margin is associated with relapse, other data have shown that the dysplastic foci at the resection margin may represent separate PanINs unrelated to the IPMN (Pflüger et al., 2020).

Understanding the genetics of precursor lesions will potentially provide helpful guidance in targeting the tumors at an earlier stage and enabling new and more effective therapeutic approaches. These could prevent malignant transformation or perhaps treat cancers arising from IPMNs. Discovering the *KRAS* mutation as a driver in most IPMNs has identified potential targeted therapies with novel inhibitors and antibodies that target mutant *KRAS* (Arbour et al., 2021; Douglass et al., 2021). Additionally, the therapies that inhibit the *Wnt* pathway may have potential in tumors with the loss of function mutation in *RNF43* (Yu et al., 2020).

## Conclusion and Perspectives

Despite the improved understanding of the pathophysiology underlying IPMNs, IOPNs, and ITPNs over the recent years, there are still many patients with lesions that mimic IPMNs—named by some authors “pseudo-IPMNs” (Muraki et al., 2022)—who undergo unnecessary surgeries and are overtreated. New approaches to predict the risk of progression preoperatively more accurately and risk of recurrence following surgery are needed. The 5th edition of the WHO classification scheme of IPMNs with the introduction of IOPNs and ITPNs as separate entities has created new opportunities for more nuanced future studies and more targeted strategies in the clinical management of these patients.

## Author Contributions

NA and SB contributed to original draft preparation of the manuscript. JS contributed to the review design, writing, and editing. All authors reviewed and approved the final version of the manuscript.

## Funding

JS is supported in part by the National Cancer Institute of the National Institutes of Health under award number K08CA234222.

## Conflict of Interest

The authors declare that the research was conducted in the absence of any commercial or financial relationships that could be construed as a potential conflict of interest.

## Publisher’s Note

All claims expressed in this article are solely those of the authors and do not necessarily represent those of their affiliated organizations, or those of the publisher, the editors and the reviewers. Any product that may be evaluated in this article, or claim that may be made by its manufacturer, is not guaranteed or endorsed by the publisher.
