# Corrigendum: Updates in the Diagnosis of Intraductal Neoplasms of the Pancreas

**DOI:** 10.3389/fphys.2022.923917

**Published:** 2022-05-13

**Authors:** Naziheh Assarzadegan, Sepideh Babaniamansour, Jiaqi Shi

**Affiliations:** Department of Pathology, University of Michigan, Ann Arbor, MI, United States

**Keywords:** intraductal papillary mucinous neoplasm, intraductal oncocytic papillary neoplasm, intraductal tubulopapillary neoplasm, pancreatic ductal adenocarcinoma, classification

An author name was incorrectly spelled as “Sepideh Babanianmansour”. The correct spelling is “Sepideh Babaniamansour”.

The authors apologize for this error and state that this does not change the scientific conclusions of the article in any way. The original article has been updated.

